# Low mortality despite temporary liver dysfunction in severe courses of acute hepatitis E

**DOI:** 10.1007/s00508-022-02126-8

**Published:** 2022-12-22

**Authors:** David J. M. Bauer, Stephan Aberle, Anna Farthofer, David Chromy, Benedikt Simbrunner, Mattias Mandorfer, Ralf Schmidt, Michael Trauner, Robert Strassl, Florian Mayer, Heidemarie Holzmann, Thomas Reiberger

**Affiliations:** 1 Vienna Hepatic Hemodynamic Laboratory, Division of Gastroenterology and Hepatology, Department of Medicine III, Medical University of Vienna, Waehringer Guertel 18–20, 1090 Vienna, Austria; 2 Vienna HIV & Liver Study Group, Medical University of Vienna, Waehringer Guertel 18–20, 1090 Vienna, Austria; 3 Department of Virology, Medical University of Vienna, Waehringer Guertel 18–20, 1090 Vienna, Austria; 4 Department of Dermatology, Medical University of Vienna, Waehringer Guertel 18–20, 1090 Vienna, Austria; 5 Institute of Clinical Virology, Department of Laboratory Medicine, Medical University of Vienna, Waehringer Guertel 18–20, 1090 Vienna, Austria

**Keywords:** Viral hepatitis, Epidemiology, Vienna, Austria, HEV

## Abstract

**Background:**

Hepatitis E virus (HEV) infection can cause severe viral hepatitis and eventually liver failure. We aim to provide novel data on the epidemiology and the course of HEV infections from Q1/2008 to Q3/2018 at the Vienna General Hospital.

**Methods:**

Of the 88,945 people tested, we identified HEV-IgM positive (+) or HEV-PCR (+) patients and retrospectively collated information on the course of infection from patient records.

**Results:**

Among 151 HEV-IgM or PCR (+) (median age 51 years, 45.8% female), 7 (4.6%) had non-severe acute HEV infection (ALT ≥ 2–5-fold upper limit of normal, ULN), 11 (7.3%) had severe HEV infection without liver dysfunction (LD) (ALT > 5-fold ULN), and 9 (6.0%) with LD (ikterus or bilirubin > 5 mg/dL, OR coagulopathy or INR > 1.5, OR encephalopathy or ammonia > 100 µmol/L). HEV-RNA-PCR was performed in 58/190 (30.5%) HEV-IgM (+) patients and was positive in 19 (30.6%). Rates of HEV IgM/PCR positivity remained stable over the observation period.

The HEV genotype (GT) was GT‑1 in 71.4% (*n* = 5) and GT‑3 in 28.6% (*n* = 2). Travel history was recorded for 9/20 (45.0%) of severe HEV and 12/20 (60.0%) patients with severe HEV infection were hospitalized. One patient with pre-existing liver disease and concomitant EBV infection required intensive care. No patient required transplantation and the 30-day mortality was 3/151 (1.9%). Despite the increased testing rates, the absolute number of diagnosed HEV infections at Vienna General Hospital remained constant between 2008 to 2018.

**Conclusion:**

Although approximately half of the patients with severe acute HEV infection required hospitalization, admissions to the intensive care unit (ICU) and short-term mortality were low.

**Supplementary Information:**

The online version of this article (10.1007/s00508-022-02126-8) contains supplementary material, which is available to authorized users.

## Introduction

Reports of hotspots and the clinical outcomes of hepatitis E virus (HEV) infections have recently raised concern internationally, particularly in Europe [1]. The annual incidence of hepatitis E worldwide is estimated at 20 million cases of infections, with 3.3 million cases of symptomatic hepatitis E, leading to global mortality of 44,000 deaths in 2005 [2].

In most immunocompetent individuals, HEV infection leads to self-limiting acute viral hepatitis, which is clinically indistinguishable from other viral acute forms of hepatitis. Similar to most other hepatotropic viruses, acute HEV is asymptomatic in most cases [3, 4]; however, HEV infection can cause extrahepatic manifestations such as neurological (including Guillain–Barré syndrome, neuralgic amyotrophy, and encephalitis), cardiovascular, hematological (including thrombocytopenia and hemolysis), and gastrointestinal (e.g., pancreatitis) complications. Higher rates of HEV immunoglobulin G (IgG) positive (+) among patients with autoimmune hepatitis (AIH) compared to the normal population suggest HEV as a possible trigger for AIH [5].

In more than 65% of acute HEV infections in solid organ recipients, HEV infection becomes chronic [6], often causing liver fibrosis and cirrhosis. In patients with pre-existing liver disease (i.e., hepatitis B), an HEV superinfection accelerates disease progression and increases mortality [7].

Overall, eight genotypes of HEV are known and GT‑3 is the most prevalent among autochthonous infections in Europe and other high-income regions, while sporadic travel-related infections with the other GT have been reported [8–11].

In developing countries, HEV is mainly transmitted by contaminated drinking water [12], while zoonotic transmission by undercooked or uncooked meat (e.g., pork and game) is common in developed countries [13]. HEV can also be transmitted vertically, from mother to child, or rarely by blood products [14]. Importantly, if both mother and fetus are infected with HEV, eclampsia, hemorrhagic complications, and fulminant liver failure may occur, mainly associated with HEV GT‑1 and 2 and predominantly during the third trimester [15].

Within Europe, several hotspots of HEV infection have been identified. These include but are not limited to southwest France [16], Scotland [17], west-central Poland [18], and the Czech Republic [19]. Although no unifying reason for hotspot formation has been established yet, local dietary habits, such as the consumption of uncooked specialty foods, may play a role. While the information on the epidemiology of HEV in Austria is available, recent information and data on its clinical course are sparse. Selective screening of high-risk Austrian blood donors from 2016 to 2017 found 29 of 155,691 (0.00019%) HEV polymerase chain reaction (PCR) (+) [20], while in a screening of the general blood donor pool in Upper Austria in 2015 7 of 58,915 (0.00012%) were HEV PCR (+) ||| and 13.6% were HEV-IgG (+) [21].

Therefore we set out to identify patients with likely acute HEV infection from all people who tested positive for HEV immunoglobulin M (IgM) or PCR in a large tertiary center and describe their characteristics, clinical course, and frequency of specific symptomes, especially among those with severe hepatitis and those showing signs of liver dysfunction.

## Patients and methods

### Study cohort

We identified patients who were tested for HEV IgG, IgM or PCR (+) between 1 January 2008, and 21 September 2018, at Vienna General Hospital. Their electronic patient records were analyzed for standard laboratory parameters, including alanine transaminase (ALT) at the date of HEV IgM or PCR (+) testing ±7 days. Patients without recorded ALT (lost to follow-up), or who were HEV-PCR negative (−), or people under 18 years of age at the time of the test were excluded.

### Clinical parameters and follow-up

The mode of collection of laboratory and clinical parameters and the follow-up are described in detail in the supplementary methods.

### Case definitions

To stratify patients according to the probability of HEV infection and the severity of the disease, we divided them into the following groups:

1. Isolated HEV-IgM (+): persons who were isolated HEV-IgM (+) but did not show ALT > 2 times the gender-specific upper limit of normal (ULN), were not counted as likely HEV infection, because in patients without other signs of liver disease, the chance of (unspecific) false positive testing is high. As HEV-IgM is not performed in asymptomatic patients outside the academic setting, it would not represent a comparable and thus useful study population.
2. Non-severe acute HEV infection: patients who were HEV-IgM or PCR (+) and had ALT elevation 2–5 times ULN.
3. Severe acute HEV: HEV-IgM or PCR (+) patients with ALT ≥ 5 times the ULN. Among patients with acute severe HEV, we differentiated patients without and with liver dysfunction (LD), where LD was defined by the presence of at least one of the following: i) jaundice or serum bilirubin > 5 mg/dL, ii) coagulopathy or International Normalized Ratio (INR) > 1.5 in the absence of oral anticoagulant therapy or iii) hepatic encephalopathy or serum ammonia (NH_3_) > 100 µmol/mL Patients tested HEV-PCR (−) were only considered in the analysis of positivity rates in HEV-PCR tests. Chronic HEV infection was defined as the persistence of HEV-PCR (+) for ≥ 3 months, according to published definitions [22].

### Virus testing and statistical analysis

The testing methods for HEV IgM, IgG, and PCR as well as coinfection with other hepatotropic viruses and the statistical analysis methods are described in the Supplementary Material. The ethics statement is also contained there.

## Results

**Fig. 1:**
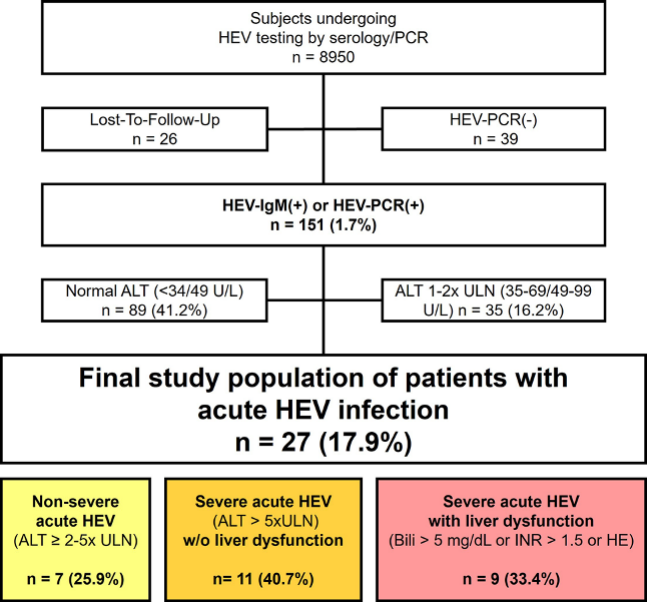
Study flowchart. *HEV* Hepatitis E Virus, *IgM* Immunoglobulin M, *PCR* polymerase chain reaction, *(+)* positive, *(–)* negative, *ULN* (gender-specific) upper limit of normal, *w/o* without, *Bili* bilirubin, *HE* hepatic encelopathy, *INR* international normalized ratio, *n* number

We retrospectively identified 8945 patients who were tested for HEV-IgM or HEV-PCR. All persons tested for HEV-IgM were also tested for IgG. Of these, 2602 (29.1%) tested HEV-IgG (+), 211 (2.4%) tested HEV-IgM (+), and 5 tested only HEV-PCR (+), 39 of 216 (18.1%) were HEV-PCR negative (−) and 26 of 216 (12.0%) HEV-IgM or PCR (+) met exclusion criteria (one patient who was solely HEV (+) did not receive any other laboratory testing and no clinical documentation was available and so was not further considered). Therefore, finally, 151 patients were included. 124 of 151 (82.1%) were isolated HEV-IgM (+), while 89 of 124 (71.8%) isolated HEV-IgM (+) did not have an elevation in ALT, and 35 of 124 (28.2%) had an elevation in ALT of 1–2 times ULN. Among the remaining 27 of 151 (17.9%) cases with an elevation of ALT ≥ 2 times ULN, we identified 7 of 27 (25.9%) non-severe cases of acute HEV infection (ALT elevation of ≥ 2–5 × ULN) and 20 of 27 (74.1%) cases of severe acute HEV infection (≥ 5 × ULN), including 9 of 27 (33.4%) patients who developed LD. The study flow chart is shown in Fig. 1.

### Demographic, clinical, and laboratory characteristics of HEV cases

HEV-IgM or PCR (+) persons were 51 [median, IQR: 27.3] years old and predominantly male (84 of 151; 55.6%). The presence of moderate ascites (i.e., 2 Child-Pugh-Turgot score [CPS] points) was recorded in each 1 of 7 (14.3%) patients with non-severe HEV and 1 of 11 (9.1%) with severe HEV w/o (other) LD, who both suffered from a chronic liver disease other than HEV infection. Mild hepatic encephalopathy (HE) was recorded in the above mentioned patient with non-severe acute HEV and in no patients with severe acute HEV infection. The patient characteristics of acute HEV cases by the severity of HEV infection are shown in Table 1.

**Table 1:** General patient characteristics. *P*-values, comparing non-severe acute HEV vs. severe HEV w/o LD vs. severe HEV w LD

	Non-severe acute HEV, *n* = 7		Severe HEV w/o liver dysfunction, *n* = 11		Severe HEV w liver dysfunction, *n* = 9		*p*‑value
Age, years [IQR]	45	[25]	39	[33]	36.5	[32.8]	0.833
Sex, *n* (%)							0.522
Male	5	(71.4%)	7	(77.8%)	7	(77.8%)	
Female	2	(28.6%)	4	(22.2%)	2	(22.2%)	
Hospital admission, *n* (%)	3	(50%)	4	(36.4%)	8	(88.8%)	0.055
ICU admission, *n* (%)	0	(0%)	0	(0%)	0	(0.0%)	–
Ascites, CP points (%)							–
1	5	(83.3%)	10	(90.9%)	9	(100.0%)	
2	1	(16.7%)	1	(9.09%)	0	(0%)	
3	0	(0%)	0	(0%)	0	(0%)	
Hepatic encephalopathy, CP points (%)							–
1	5	83.3	11	(100%)	9	(100%)	
2	1	(16.7%)	0	(0%)	0	(0%)	
3	0	(0%)	0	(0%)	0	(0%)	
LTX evaluated, *n* (%)	2	(33.3%)	0	(0%)	0	(0%)	**0.027**
Travel history, *n* (%)	0	(0%)	3	(27.3%)	6	(66.7%)	***0.023***
LTX, *n* (%)	2	(33.3%)	1	(9.1%)	0	(0%)	0.133
30-day mortality, *n* (%)	2	(50%)	0	0%	0	0%	–
–	**Laboratory values at diagnosis of HEV**						
Dg Hb, g/dL, mean ± SD	14.0	±1.6	14.9	±0.9	13.7	±1.9	0.267
Dg PLT, G/L, median [IQR]	176	[129]	268	[127]	202	[110]	0.230
Dg WBC, G/L, median [IQR]	5.5	[3.6]	7.0	[2.6]	6.7	[2.8]	0.525
Dg Na, mmol/L, median [IQR]	141	[2]	139	[4]	138	[2]	0.074
Dg Creatinine, mg/dL, median [IQR]	0.85	[0.22]	0.87	[0.13]	0.92	[0.17]	0.483
Dg Bilirubin, mg/dL, median [IQR]	1.0	[2.4]	1.1	[0.8]	9.4	[4.0]	***0.001***
Dg Albumin, g/L, median [IQR]	35.4	[11.6]	44.0	[7.8]	35.0	[4.9]	***0.045***
Dg ALP, U/L, median [IQR]	98	[202]	191	[94]	158	[52]	*0.394*
Dg AST, U/L, median [IQR]	119	[171]	336	[481]	1535	[2419]	**0.001**
Dg ALT, U/L, median [IQR]	140	[47]	523	[946]	2245	[1598]	*<* **0.001**
Dg gGT, U/L, median [IQR]	156	[139]	190	[248]	138	[163]	*0.558*
Dg NH_3_, mmol/L, median [IQR]	56.4	[38.6]	26.3	[6.0]	34.1	[23.9]	0.946
Dg INR, median [IQR]	1.1	[0.1]	1.1	[0.2]	1.6	[0.0]	**0.011**
–	**Laboratory values at ALT peak**						
Peak Hb, g/L, mean ± SD	13.1	±2.1	14.0	±1.8	15.2	±1.1	0.039
Peak PLT, G/L. mean ± SD	209	±112	214	±68	210	±79	0.944
Peak WBC, G/L, median [IQR]	5.0	[4.7]	6.8	[2.5]	7.7	[3.7]	0.359
Peak Na, mmol/L, median [IQR]	140	[2.25]	137	[3]	138	[2.5]	0.290
Peak Creatinine, mg/dL, median [IQR]	0.82	[0.21]	0.87	[0.22]	0.87	[0.27]	0.825
Peak BUN, mg/dL, median [IQR]	11	[2]	12	[4]	11	[5]	0.434
Peak Bilirubin, mg/dL, median [IQR]	1.7	[2.4]	1.2	[2.8]	9.4	[6.5]	***0.002***
Peak Albumin, g/L, mg/dL, median [IQR]	37.9	[6.4]	36.2	[5.0]	43.7	[4.2]	*0.099*
Peak ALP, U/L, mean ± SD	237	± 181	202	± 109	225	± 79	0.841
Peak AST, U/L, mean ± SD	228	± 49	572	± 679	2424	± 1996	***0.001***
Peak ALT, U/L median [IQR]	1867	[230]	1310	[1188]	2819	[1094]	***<*** ***0.001***
Peak GGT, U/L, mean ± SD	122	± 231	261	±176	200	±169	0.510
Peak INR, mean ± SD	1.2	±0.3	1.2	±0.2	1.6	±0.2	***0.021***
Peak NH_3_, mmol/L, mean ± SD	–	–	28.4	±3.0	33.5	±17.1	0.718

*ALP* alkaline phosphatase, *ALT* alanine transaminase, *AST* aspartate transaminase, *CP points* Child-Pugh points, *Dg* at the time of diagnosis of HEV ± 7 days, *gGT* gamma-glutamyl transferase, *Hb* hemoglobin, *ICU* intensive care unit, *INR* international normalized ratio, *IQR* interquartile range, *LTX* liver transplant, *N* number, *Na* sodium, *NH*_*3*_ ammonia, *Peak* laboratory values at ALT peak, *PLT* platelets, *SD* standard deviation, *WBC* white blood cells

### Laboratory parameters at diagnosis

Median serum hemoglobin at diagnosis of HEV (Dg) increased with severity of HEV infection (IgM (+): median: 13.2 [IQR: 3.2] vs. severe HEV w/o LD: 13.7 [1.7] vs. severe HEV w LD: 14.6 [1.8] g/dL, *p* = 0.004), as did median Dg alkaline phosphatase (ALP) (median: 84 [49] vs. 191 [94] vs. 158 [52] U/L, *p* < 0.001), Dg gamma-glutamyl transferase (gGT) (median: 40 [81] vs. 190 [248] vs. 138 [163], *p* < 0.001), Dg INR (median: 1.1 [0.2] vs. 1.1 [0.2] vs. 1.6 [0], *p* = 0.011), Dg Bilirubin (median: 0.6 [0.5] vs. 1.1 [0.8] vs. 9.4 [4.0] mg/dL, *p* < 0.001), Dg AST (median: 28 [16] vs. 336 [481] vs. 1535 [2419] U/L, *p* < 0.001) and Dg ALT (30 [31] vs. 523 [946] vs. 2245 [1598] U/L, *p* < 0.001). In contrast, serum albumin was lower in patients with more severe disease (median: 41.6 [IQR: 9.1] vs. 44.0 [7.8] vs. 35.0 [4.9] g/L, *p* = 0.013). Baseline characteristics of isolated HEV-IgM (+) are shown in Supplemental Table-ST1.

### Laboratory parameters at peak

Laboratory parameters at ALT peak were evaluated for people with non-severe acute HEV and severe HEV. Serum levels of AST and ALT increased with the severity of hepatitis: AST (mean: non-severe HEV: 228 [49] vs. HEV w/o LD: 572 [679] vs. HEV w LD: 2424 [1996], *p* = 0.001) and ALT (median: 187 [230] vs. 1310 [1188] vs. 2819 [1094], *p* < 0.001), while levels of bilirubin (median: 1.7 [2.4] vs. 1.2 [2.8] vs. 9.4 [6.5], *p* = 0.002) and INR (mean: 1.2 ± 0.2 vs. 1.6 ± 0.2, *p* = 0.005) were higher, when comparing HEV w/o LD to HEV w LD.

**Fig. 2:**
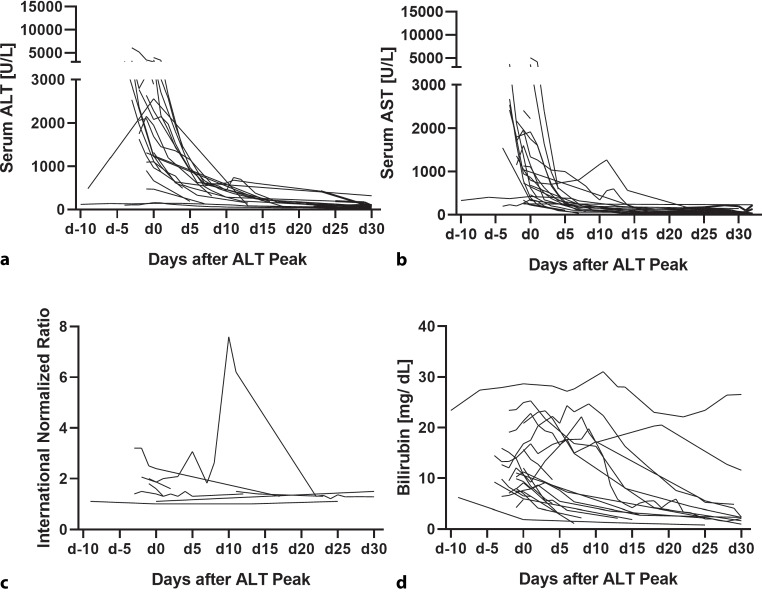
The course of **a** ALT, **b** AST, **c** INR, and **d** bilirubin in patients with severe HEV with liver dysfunction (LD)

In severe HEV, the ALT peak occurred 1 day [IQR: 3.25 days] before the first IgM or PCR (+), while the AST peak occurred on that day of positivity [IQR: 0.5 days]. However, bilirubin and INR peaks occurred on the day of the first HEV-IgM or PCR (+), but the occurrences were more widely spread [IQR: 0 and IQR: 4.5 days]. The time course of these laboratory parameters in patients with LD is shown in Fig. 2.

Of the 18 patients with non-severe acute HEV and severe HEV w/o LD, 1 (5.6%) developed LD within 30 days.

### HEV genotype

HEV-GT was available in 7 patients, 5 (71.4%) of whom were infected with GT‑1 and 2 (28.6%) with GT‑3. We did not find a statistically significant difference in GT rates between severity groups. 4 out of 5 (80.0%) patients with GT‑1 had travelled to endemic areas, while one of the GT-3 patients had travelled within Europe. None of the patients with severe HEV was pregnant at the time of infection. We assessed for but did not find any coinfection with HBV, HCV, or HIV amongst patients with severe HEV (either w/o or w LD)—see Supplemental Table-ST2.

### Testing rates

Considering the HEV IgM (+) including *n* = 39 HEV-PCR (−), 58 of 190 (30.5%) HEV-IgM (+) were tested for HEV-RNA at the time of diagnosis or retrospectively from stored samples. In total, 19 of 58 (32.8%) were HEV-PCR (+). The distribution of HEV-PCR (+) among the subgroups, the proportion of HEV-PCR (+) tests, the median concentration of HEV-RNA, and the distribution of HEV-genotype (GT) by group are shown in Supplemental Table-ST3.

**Fig. 3:**
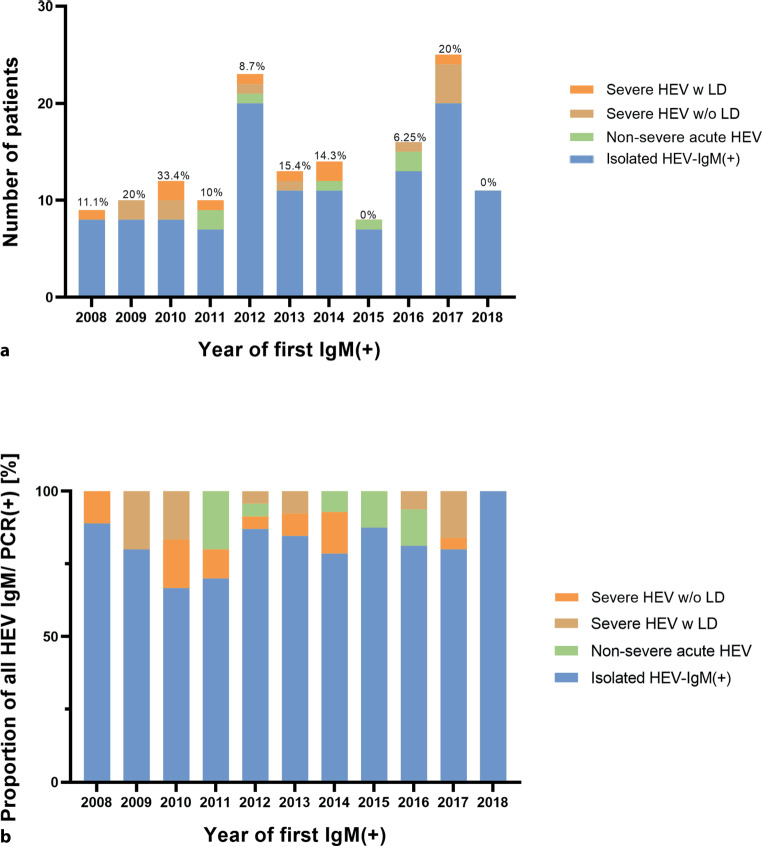
Number of HEV-IgM (+) per year. Stacked bar chart showing the number of HEV-IgM-positive (+) and proportion of isolated HEV-IgM (+), non-severe, severe HEV with (w) and without (w/o) liver dysfunction (LD) per year. **a** Number of HEV-IgM/PCR (+) patients from 2008–2018, with the cumulative percentage of severe HEV w LD (orange), w/o LD (brown) and non-severe acute HEV (green) of the total given above the bar **b** proportion of HEV-IgM or PCR (+) per year

The number of HEV-IgM tests performed increased steadily from 2008 to 2017 (the number was lower in 2018, but only 3 quarters of the year 2018 were evaluated), while the HEV-IgM or PCR (+) generally decreased with a transient increase in 2015. However, the proportion of severe HEV infection was highest in 2009, 2010, and 2017, as shown in Fig. 3.

Annual male to female (M:F) ratio of HEV-IgM or PCR (+) undulated between 0.43 (M *n* = 3/F *n* = 7) in 2009 and 3 (M *n* = 6/F *n* = 2) in 2015. The numbers for Fig. 2 are supplied in Supplemental Table-ST4.

### Risk factors for HEV transmission

Travel anamnesis was documented in 3 of 11 (27.3%) severe HEV w/o LD and 6 of 9 (66.7%) severe HEV w LD. We could not identify any men who have sex with men (MSM) from the records among patients with non-severe HEV and w/o or w LD, although this might be due to a lack of documentation.

### Symptom frequency

Symptoms other than hepatic encephalopathy and ascites were recorded in 10 of 11 (90.9%) and 8 of 9 (88.9%) patients with severe HEV w/o and w LD. Most commonly noted symptoms were jaundice (w/o LD: 2 of 10 (20.0%) vs. w LD: 7 of 8 (87.5%)), dark urine (4 of 10 (40.0%) vs. 7 of 8 (87.5%)), abdominal pain/discomfort (4 of 10 (40.0%) vs. 6 of 8 (75.0%)), nausea (w/o LD: 5 of 10 (50.0%) vs. w LD: 2 of 8 (25.0%)), weight loss (3 of 10 (30.0%) vs. 6 of 8 (75.0%)), emesis/vomiting (2 of 10 (20.0%) vs. 2 of 8 (25.0%)), and fever (4 of 10 (40.0%) vs. 4 of 8 (50.0%)). Diarrhea, hepatomegaly, and deteriorated general condition (DGC)/fatigue and malaise were also frequently recorded. Compare with Table 2.

**Table 2:** Case symptom frequency in severe HEV; in patients without (w/o) and with (w) liver dysfunction; *P*-values, comparing non-severe acute HEV vs. severe HEV w/o LD vs. severe HEV w LD

Symptom frequency							
Symptoms, *n* (%)	Non-severe acute HEV, *n* = 7		Severe HEV w/o LD *n* = 11		Severe HEV w LD *n* = 9		*p*-value
Jaundice	1	14.3%	2	20.0%	7	87.5%	**0.016**
Dark urine	–	–	4	40.0%	7	87.5%	**0.02**
Abdominal discomfort/pain	1	14.3%	4	40.0%	6	75.0%	0.348
Weight loss	–	–	3	39.0%	6	75.0%	**0.043**
Emesis	–	–	2	20.0%	2	25.0%	1
Fever	1	14.3%	4	40.0%	4	50.0%	1
Acholic stool	–	–	3	30.0%	2	25.0%	0.170
Diarrhea	–	–	4	40.0%	2	25.0%	0.551
Fatigue/malaise	1	14.3%	5	50.0%	3	37.5%	0.85
Night sweats	–	–	–	–	5	62.5%	**0.007**
Nausea	–	–	5	50.0%	2	25.0%	0.269
Pruritus	–	–	1	10.0%	1	12.5%	1
Exanthema	–	–	1	10.0%	–	–	1
Arthralgia	1	14.3%	1	10.0%	–	–	0.405
Deteriorated general condition (DGC)	1	14.3%	3	30.0%	2	25.0%	1
Symptoms recorded	**3 (42.9%)**		***n*** **=** **10 (90.9%)**		***n*** **=** **8 (88.8%)**		–

*DGC* deteriorated general condition, *N* number, *w* with, *w/o* without

### Clinical course and outcomes of severe HEV infections

Pre-existing liver disease was a disease-modifying factor in 2 of 20 (10.0%) patients who had severe HEV, while immunosuppression (i.e., due to previous non-liver organ transplantation, concomitant other viral or bacterial infection, diabetes mellitus, or autoimmune disease) was observed in 7 of 20 (35.0%) patients with severe HEV. A description of the clinical characteristics and disease course of patients with severe HEV is shown in Supplemental Table-ST5.

Out of 20 patients with severe HEV infection 12 (60.0%) were admitted to the hospital, of which 4 (33.4%) had no LD and 8 (66.7%) had LD.

The 30-day mortality, as assessed by a query to the hospital records as well as to the national registry of deaths, showed a low mortality rate (IgM (+): 3 of 131 (2.3%) severe HEV w/o and w LD: 0 of 20. One patient who had pre-existing liver disease and died on the ICU after contracting EBV and becoming HEV-IgM-positive, was not counted towards severe HEV w LD, as etiological factors other than HEV-infection might have been leading. Their clinical course is described in the supplementary materials. The other 2, who died within 30 days, died of non-liver-related causes, and were affected by severe systemic diseases (influenza requiring invasive ventilation and non-liver metastasized carcinoma) at the time of HEV-IgM testing.

A test of cure showing negative stool HEV-PCR was performed in 4 of 58 (6.9%) PCR (+). However, in 7 of 58 (12.1%), HEV-PCR was positive in the last stool PCR testing, indicating that our center did not perform a test of cure in these patients.

### Prolonged HEV viremia and relapsing/chronic course

Two patients who had previously received a solid organ transplant developed prolonged HEV viremia and were treated with an adaption of immunosuppression and ribavirin. No cholestatic courses of HEV infection amongst patients with non-severe or severe acute HEV infection were observed (see Supplemental Figure-SF1).

### Extrahepatic manifestations

No extrahepatic manifestations were found in a review of the clinical documentation of the cases of acute HEV infection identified here. Possible extrahepatic manifestations of HEV infection were documented in a minority of cases: 3/20 (15.0%) patients developed anemia with concordantly elevated serum lactate dehydrogenase during the suspected HEV infection. However, in none of these cases, was the clinical diagnosis of hemolytic anemia made, or a transfusion of blood products was necessary. While only for each 1 of 20 (5.0%) patients with severe HEV infection, arthralgia or exanthema were documented (see Supplemental Table-ST6).

## Discussion

In this retrospective study, we describe the epidemiology, clinical characteristics, and disease course of HEV-IgM (+) patients, at a large tertiary center in central Europe between 2008 and 2018. We defined an elevation in ALT ≥ 2–5 times the ULN as acute non-severe HEV infection, an elevation in ALT > 5 times the ULN as acute severe HEV infection, and liver dysfunction as clinical or laboratory signs of 1) coagulopathy, 2) jaundice, or 3) hepatic encephalopathy.

Of the 8945 people tested for HEV, 2520 (28.2%) were anti-HEV-IgG (+), implying that 28.2% of patients had previous contact with the HEV virus. This is in line with reports for other European countries ranging from 2.2% in Madrid, Spain, [23] to 52.5% in hotspot regions, such as southwestern France [24], but higher than the 13.6% HEV-IgG (+) rate reported from blood donor screening of the Austrian general population [21]. In this respect, it is essential to note that this number reflects the IgG seropositivity amongst a selected population of presumably symptomatic patients at a tertiary center. All 8945 patients were tested for HEV-IgM and IgG. This equal number of tests is due to the laboratory request form’s design, where HEV-IgG and IgM can only be requested together. Interestingly, more men (84 men vs. 67 women) were tested for HEV-IgM or -PCR (+), while the male to female ratio changed without a discernible pattern during the observation period.

Overall, we observed stable numbers of HEV infections—despite increasing numbers of HEV-IgM and IgG tests—and stable numbers of severe disease courses without and with liver dysfunction during the study period. This increase in HEV-IgM and IgG tested individuals is comparable to the increase in testing for other hepatotropic viruses at our center (e.g., HAV) [25]. The sensitivity and specificity especially of the serological HEV tests used increased over the observation period. Whether a constant absolute rate of HEV seropositivity in conjunction with increased accuracy indicates a higher rate of infections (this is assuming fewer false positives) remains speculation. The relative rates of severe HEV infection were higher in 2009, 2010, and 2017. However, no clear temporal or epidemiological trends were found, and most acute HEV infections were documented to be travel-associated.

Biochemical alterations associated with acute HEV reportedly persist for 4–6 weeks, while symptoms resolve after 1–2 weeks [26]. In contrast in our cohort transaminases normalized within 10 days after the peak of ALT peak (Fig. 2a,b). Bilirubin also normalized faster than previously reported in most patients, although, in some patients, bilirubin normalization took up to 1 month (Fig. 2d).

To characterize the clinical course of HEV infection, we assessed the recorded symptoms of patients with severe hepatitis E, finding a spectrum consistent with previous reports and reviews [3, 27] (Compare Table 2). No cases of extrahepatic manifestations particularly thrombocytopenia, kidney injury, hemolytic anemia, pancreatitis, and neurological symptoms were documented (compare Supplementary Table ST7), likely due to a combination of the relative rarity of these symptoms [28] and the retrospective nature of our approach. The frequency of jaundice was comparable to other reports [4, 26].

The HEV-IgM test is often unspecifically (i.e., falsely) positive, as demonstrated by the high rates of HEV-IgM (+), but HEV-PCR (−) patients without transaminase elevation. Unspecific (false positive) HEV-IgM tests are also an issue in critically ill patients, as there is a considerably higher rate of HEV-PCR negativity in patients with non-severe and even amongst those with severe hepatitis (compare Supplemental Table-ST1). Therefore, positive serological HEV results should always be verified using HEV-PCR because persons who are severely ill or are otherwise immunocompromised or immunosuppressed are more likely to contract HEV and subsequently develop clinically symptomatic HEV infection [29]. Inline, 2 of the chronically ill patients were under immunosuppressive medication due to previous solid organ transplants and were treated with the adaptation of immunosuppression and ribavirin after chronification of HEV. Although one patient cleared HEV after 5 months, the other patient remained low-level HEV viremic. Both clinical courses are described in more detail in the supplementary material.

Liver transplantation is a treatment for liver failure due to HEV infection. In our cohort, 2 patients who were HEV-IgM (+), without severe HEV infection, were evaluated for liver transplantation but ultimately none received liver transplantation within 30 days. Of importance, the driving factor for the evaluation of liver transplantation in these patients was not HEV infection but other pre-existing liver diseases. Both cases are discussed in more detail in the supplementary material.

The present study is a retrospective study, and the data are based on clinical documentation at the time of suspected HEV infection. The rates of symptoms and clinical characteristics may be underdocumented and therefore underestimated. The fact that this study was carried out in a large tertiary center is a limitation in that it might show higher rates of prevalence and symptoms than in the general population, as symptomatic and vulnerable people are more likely to be referred to a tertiary center. However, this is also a strength as it allows the identification of a greater number of symptomatic and compromised patients, enabling a more robust description of characteristics of interest of the rare Austrian cases of acute HEV infections.

To summarize, we identified 216 HEV-IgM or PCR (+) patients at the General Hospital of Vienna between 2008 and 2018. Annual rates of acute HEV infections remained stable despite increased testing. Biochemical aberrations due to severe hepatitis E resolved primarily within 1–2 weeks. Two patients under immunosuppression due to a history of solid organ transplantation developed prolonged viremia after acute HEV infections, and both were treated with an adaptation of immunosuppression and ribavirin, 20 patients developed severe HEV infection and, of these, 9 developed liver dysfunctions.

In conclusion, despite increasing testing, we observed stable numbers of acute HEV infections in Vienna in the last decade with a low risk of short-term liver-related morbidity and mortality.

## Supplementary Information

The Supplementary Information contains a description of the collection of clinical, laboratory parameters, virological testing, statistical analysis, as well as the ethical apporval statement and descriptions of the clinical course of patients of interest mentioned in the main text. Additionally the Supplemental Tables refered to in the main text, concerning the baseline characteristics of HEV IgM(+), coinfection rates, testing rates, male to female ratio of severe HEV, patient characteristics of severe HEV, as well as extrahepatic manifestation rates and graphs showing the time course of gGT and ALP in severe HEV, are contained therein

## Abbreviations

AIH: Autoimmune hepatitis
ALP: Alkaline phosphatase
ALT: Alanine transaminase
ANOVA: Analysis of variance
AST: Aspartate transaminase
CPS: Child-Pugh-Turgot score
Dg: Diagnosis
DGC: Deteriorated general condition
EBV: Epstein-Barr virus
gGT: Gamma-glutamyl transferase
HAV: Hepatitis A virus
HBV: Hepatitis B virus
HCV: Hepatitis C virus
HEV: Hepatitis E virus
HIV: Human immunodeficiency viruses
ICU: Intensive care unit
IgG: Immunoglobulin G
IgM: Immunoglobulin M
INR: International normalized ratio
IQR: Interquartile range
LDH: Lactate dehydrogenase
MSM: Men who have sex with men
NH3: Serum ammonia
PCR: Polymerase chain reaction
RNA: Ribonucleic acid
ULN: Gender-specific upper limit of normal
w LD: With liver dysfunction
w/o LD: Without liver dysfunction

## References

[1] The Lancet (2017). Growing concerns of hepatitis E in Europe. Lancet.

[2] Rein DB, Stevens GA, Theaker J, Wittenborn JS, Wiersma ST (2012). The global burden of hepatitis E virus genotypes 1 and 2 in 2005. Hepatology.

[3] Aslan AT, Balaban HY (2020). Hepatitis E virus: epidemiology, diagnosis, clinical manifestations, and treatment. World J Gastroenterol.

[4] Said B, Ijaz S, Kafatos G, Booth L, Thomas HL, Walsh A (2009). Hepatitis E outbreak on cruise ship. Emerg Infect Dis.

[5] Eder M, Strassl R, Beinhardt S, Stättermayer AF, Kozbial K, Lagler H (2019). High seroprevalence of anti-Hepatitis E antibodies in Austrian patients with autoimmune hepatitis. Liver Int.

[6] Kamar N, Garrouste C, Haagsma EB, Garrigue V, Pischke S, Chauvet C (2011). Factors associated with chronic hepatitis in patients with hepatitis E virus infection who have received solid organ transplants. Gastroenterology.

[7] Tseng TC, Liu CJ, Chang CT, Su TH, Yang WT, Tsai CH (2020). HEV superinfection accelerates disease progression in patients with chronic HBV infection and increases mortality in those with cirrhosis. J Hepatol.

[8] Mansuy JM, Peron JM, Abravanel F, Poirson H, Dubois M, Miedouge M (2004). Hepatitis E in the south west of France in individuals who have never visited an endemic area. J Med Virol.

[9] Dalton HR, Stableforth W, Thurairajah P, Hazeldine S, Remnarace R, Usama W (2008). Autochthonous hepatitis E in Southwest England: Natural history, complications and seasonal variation, and hepatitis e virus IgG seroprevalence in blood donors, the elderly and patients with chronic liver disease. Eur J Gastroenterol Hepatol.

[10] Wichmann O, Schimanski S, Koch J, Kohler M, Rothe C, Plentz A (2008). Phylogenetic and case-control study on hepatitis E virus infection in Germany. J Infect Dis.

[11] Kamar N, Bendall R, Legrand-Abravanel F, Xia NS, Ijaz S, Izopet J (2012). Hepatitis E. Lancet.

[12] Dalton HR, Bendall R, Ijaz S, Banks M (2008). Hepatitis E: an emerging infection in developed countries. Lancet Infect Dis.

[13] Slot E, Zaaijer HL, Molier M, Van Den Hurk K, Prinsze F, Hogema BM (2017). Meat consumption is a major risk factor for hepatitis E virus infection. PLoS ONE.

[14] Bi H, Yang R, Wu C, Xia J (2020). Hepatitis E virus and blood transfusion safety. Epidemiol Infect.

[15] Khuroo MS, Kamili S (2003). Aetiology, clinical course and outcome of sporadic acute viral hepatitis in pregnancy. J Viral Hepat.

[16] Mansuy JM, Sauné K, Rech H, Abravanel F, Mengelle C, L’Homme S (2015). Seroprevalence in blood donors reveals widespread, multi-source exposure to hepatitis E virus, southern France, October 2011. Euro Surveill.

[17] Thom K, Gilhooly P, McGowan K, Malloy K, Jarvis LM, Crossan C (2018). Hepatitis E virus (HEV) in Scotland: evidence of recent increase in viral circulation in humans. Euro Surveill.

[18] Bura M, Łagiedo M, Michalak M, Sikora J, Mozer-Lisewska I (2017). Hepatitis E virus IgG seroprevalence in HIV patients and blood donors, west-central Poland. Int J Infect Dis.

[19] Adlhoch C, Avellon A, Baylis SA, Ciccaglione AR, Couturier E, de Sousa R (2016). Hepatitis E virus: assessment of the epidemiological situation in humans in Europe, 2014/15. J Clin Virol.

[20] Boland F, Martinez A, Pomeroy L, O’Flaherty N (2019). Blood donor screening for hepatitis E virus in the European Union. Transfus Med Hemother.

[21] Fischer C, Hofmann M, Danzer M, Hofer K, Kaar J, Gabriel C (2015). Seroprevalence and incidence of hepatitis E in blood donors in Upper Austria. PLoS ONE.

[22] Dalton HR, Kamar N, Baylis SA, Moradpour D, Wedemeyer H, Negro F (2018). EASL clinical practice guidelines on hepatitis E virus infection. J Hepatol.

[23] Fogeda M, Avellón A, Echevarría JM (2012). Prevalence of specific antibody to hepatitis E virus in the general population of the community of Madrid. Spain J Med Virol.

[24] Mansuy JM, Bendall R, Legrand-Abravanel F, Sauné K, Miédouge M, Ellis V (2011). Hepatitis E virus antibodies in blood donors, France. Emerg Infect Dis.

[25] Bauer D, Farthofer A, Chromy D, Simbrunner B, Steininger L, Schmidbauer C (2020). Recent outbreaks of severe hepatitis A virus infections in Vienna. Eur J Clin Microbiol Infect Dis.

[26] Kamar N, Dalton HR, Abravanel F, Izopet J (2014). Hepatitis E virus infection. Clin Microbiol Rev.

[27] Lhomme S, Marion O, Abravanel F, Izopet J, Kamar N (2020). Clinical manifestations, pathogenesis and treatment of hepatitis E virus infections. J Clin Med.

[28] Rawla P, Raj JP, Kannemkuzhiyil AJ, Aluru JS, Thandra KC, Gajendran M (2020). A systematic review of the extra-hepatic manifestations of hepatitis E virus infection. Med Sci.

[29] Riveiro-Barciela M, Buti M, Homs M, Campos-Varela I, Cantarell C, Crespo M (2014). Cirrhosis, liver transplantation and HIV infection are risk factors associated with hepatitis E virus infection. PLoS ONE.

