# Supplementary material for: Low mortality despite temporary liver dysfunction in severe courses of acute hepatitis E
Source: Wien Klin Wochenschr. 2022 Dec 22;135(3-4):57–66. doi: 10.1007/s00508-022-02126-8 (PMC9938023; doi:10.1007/s00508-022-02126-8)
Supplement: Supplementary file 1 — The Supplementary Information contains a description of the collection of clinical, laboratory parameters, virological testing, statistical analysis, as well as the ethical apporval statement and descriptions of the clinical course of patients of interest mentioned in the main text. Additionally the Supplemental Tables refered to in the main text, concerning the baseline characteristics of HEV IgM(+), coinfection rates, testing rates, male to female ratio of severe HEV, patient characteristics of severe HEV, as well as extrahepatic manifestation rates and graphs showing the time course of gGT and ALP in severe HEV, are contained therein [file 508_2022_2126_MOESM1_ESM.docx]

***Supplementary methods***

***Collection of clinical and laboratory parameters, as well as clinical follow up***

Standard laboratory parameters were acquired from patient records for all HEV-IgM & RNA-PCR (+) persons. For all patients with ALT > 2 times ULT, the course of ALT, aspartate transaminase (AST), serum bilirubin, INR, and serum NH_3_ was recorded 10 %patients whose ALT increased above the given thresholds before or after baseline but not at baseline, we evaluated whether the peak transaminases within 10 days before and 30 days after baseline were within the same thresholds/group as at baseline. All biochemical laboratory tests were performed by the hospital laboratory, which is ISO 15189 certified.

To characterize the clinical course of severe HEV infection, patient characteristics and symptoms - particularly hospital and intensive care admissions and discharge, (suspected) transmission route, travel history, planned liver transplantation and subsequent liver transplantation, oral anticoagulant therapy, as well as 30-day mortality - were extracted manually from patient records. To avoid the introduction of bias due to the delay between symptom onset and testing, we assessed the course of laboratory parameters up to 10 days before the diagnostic test, where available. The existence and classification of hepatic encephalopathy and ascites in clinical records and radiological reports were also considered, and the results were classified as Child-Pugh-Turgot Score (CPS) points. The Austrian government register of deaths at the national statistical agency "*Statistik Austria,"* and the hospitals' database was searched for registered deaths and time of death of all HEV-IgM or RNA-PCR (+) to calculate the 30-day mortality after first positive HEV testing. Clinical records of patients with severe acute HEV infection were manually reviewed for mention of other extrahepatic manifestations such as neurological (e.g., Guillain–Barré syndrome, neuralgic amyotrophy), dermatological manifestations (e.g., Cutaneous T-cell lymphoproliferative disorder), gastrointestinal (e.g., pancreatitis) or hematological manifestations (i.e., hemolytic anemia or thrombocytopenia). To independently assess the rate of hemolytic anemia, anemia was defined as Hb ˂ 10 mg/dL. All patients with such anemia at baseline +/- 30 days were evaluated for increased lactate dehydrogenase (LDH). If anemia and increased LDH were present, the records searched for the clinical diagnosis of hemolytic anemia. If applicable, the diagnosis was made from the records, even if it was not made at the time of treatment. Severe thrombocytopenia was defined as platelets ˂ 50 G/L. Only patients who developed hemolytic anemia or severe thrombocytopenia around the time of HEV infection were counted toward this extrahepatic manifestation. To evaluate the rate of acute pancreatitis, serum pancreas lipase was assessed. Where it was elevated, the clinical record was consulted on the matching diagnosis or clinical course.

***Virologic Testing***

HEV-PCR and genotyping were performed according to published recommendations [1], while HEV for IgM and IgG were assessed using the Wantai Hepatitis E ELISA Assay (Beijing Wantai Biological Pharmacy Enterprise Co., Ltd, Beijing, China). Coinfection with HIV (human immunodeficiency viruses), HAV (Hepatitis A virus), HBV (Hepatitis B virus), and HCV (Hepatitis C virus) was assessed, referring to the results of available antibody-testing and PCR testing. We retrospectively performed PCR and genotyping for non-severe and severe HEV patients where HEV-PCR results were initially unavailable, but stored serum/plasma samples were.

***Statistical analysis***

In descriptive statistics, mean and standard deviation are given for parametric variables, while the median and interquartile range (IQR) are given for nonparametric variables. The polarity of the variables was decided using the Shapiro-Wilk-Test. Comparisons of two groups were calculated using Student's T-Test or the Mann–Whitney U test, while comparisons of three or more groups were performed using one-way analysis of variance (ANOVA) or the Kruskal–Wallis test, each as appropriate. The Fisher's exact test or the Chi-Squared test were used for comparisons of frequencies between groups. The male-to-female ratio is the absolute number of males with a given characteristic divided by the absolute number of females with a particular characteristic in the given period. When giving a proportion of people with a given characteristic (e.g., afflicted by a symptom), the number of people where the information on the presence of the symptom was known was used as the denominator.

As HEV serology was only performed in patients presenting at the General Hospital of Vienna and was performed in a wide range of patients, while outside of the academic setting they are only ordered in patients with clinical suspicion of HEV infection, the serological results here do not reflect the disease's general incidence. These serological tests are also known to deliver false-positive results. Therefore, the numbers and characteristics of patients with isolated HEV IgM (+) were only used as the basis/ denominator for ratios or comparisons.

Statistical analysis was performed using R: language and environment for statistical computing [2] version 4.0.2, using the dplyr (Hadley Wickham, Romain François, et al., 2020), the openxlsx [4], and the tableone [5] packages and GraphPad Prism 8 (Prism Inc., Bellevue, WA, USA). Graphs were produced using GraphPad Prism 8 and MS PowerPoint (Microsoft Redmond, WA, USA).

**Ethical Approval statement**

This study was approved by the Ethics Committee of the Medical University of Vienna (No. 1968/2018) and was conducted in accordance with the Declaration of Helsinki and its amendments up to and including the 2013 amendment and the guidelines for Good Scientific Practice of the Medical University of Vienna.

**Supplementary information on clinical courses of interest**

**The clinical course of one patient with severe HEV infection requiring ICU admission**

One patient with preexisting liver cirrhosis presented with low ALT levels at the time of positive IgM testing but developed severe HEV w LD within 5 days and was admitted to the ICU and then transferred to the ICU of our hospital and finally died within 30 days. The patient developed elevated transaminases within five days but suffered from advanced chronic liver disease before contracting HEV infection and concomitant Epstein-Barr-Virus (EBV) infection, developing fulminant liver failure. He was not counted towards the severe HEV group, as the liver dysfunction and mortality cannot clearly be linked to HEV infection.

**Clinical Courses of chronic HEV infection**

One of the patients with severe HEV infection, who had previously received a heart transplant, developed chronic HEV infection and was initially treated with an adaptation of immunosuppression and ribavirin, whereupon the transaminases and viral load rapidly decreased. While the patient achieved negative HEV-PCR, HEV relapsed after discontinuing ribavirin therapy, although at a low level. HEV-RNA blood levels remained low, as did liver fibrosis to this day (data by January 2022). While HEV-RNA levels have been reported not to correlate with fibrosis progression, the levels of aminotransferases correlate with the risk of fibrosis progression [6].

A second patient experienced a prolonged HEV infection after receiving a lung transplant for end-stage lung fibrosis. They had a pre-existing advanced chronic liver disease of mixed etiology, which included drug-induced liver injury and alcoholic liver injury. HEV infection became apparent 5 months after transplantation. Although HEV-IgM and -IgG remained negative, a positive HEV-PCR showed infection with an elevation of liver enzymes and liver dysfunction with serum bilirubin levels ˃ 20mg/dL and an INR of 1.4. Promptly, therapy with an adaptation of immunosuppression and ribavirin was started. While transaminases and serum bilirubin normalized with 2.5 months, HEV negativity could only be shown 5 months after treatment initiation. However, the infection was clinically judged to have resulted in significant fibrosis. Two months later, a CMV infection occurred, ultimately leading to the patient’s demise.

**Liver transplant evaluation among HEV positive patients**

In total two patients in our cohort who were HEV-IgM (+), but did not have severe acute HEV infection were evaluated for liver transplantation:

One patient suffered from chronic HCV infection and had already received a liver transplant 5 years before HEV-IgM (+) and experienced relapse and re-cirrhosis in the transplanted liver. In that case, the HEV-IgM (+) was observed during incidental testing and only correlated to a slight increase in liver enzymes and is not discussed in the records. Therefore, we must assume that an HEV infection was deemed clinically unlikely, and the serological result was a false positive. Finally, the patient died while waiting for a retransplant, only 6 months later. The other patient also suffered infection with HCV and subsequently developed HCC in cirrhosis. At the time of HEV-IgM positivity, no increase in liver enzymes or decrease in liver function was observed, so although not explicitly so documented, HEV infection was clinically unlikely, so HEV (+) serology was not followed. The patient ultimately was transplanted and finally required re-transplant. In retrospect, these patients' serological results were likely falsely positive.

**Supplementary References**

1. Mulder AC, Kroneman A, Franz E, Vennema H, Tulen AD, Takkinen J, et al. HEVnet: a One Health, collaborative, interdisciplinary network and sequence data repository for enhanced hepatitis E virus molecular typing, characterisation and epidemiological investigations. Eurosurveillance. European Centre for Disease Prevention and Control (ECDC); 2019;24:1800407.

2. R Core Team. R: A language and environment for statistical computing. Cambridge; 2020.

3. Hadley Wickham, Romain François LH and KM, Wickham H, François R, Henry L, Müller K, Hadley Wickham, Romain François LH and KM. dplyr: A Grammar of Data Manipulation. 2020.

4. Walker A. openxlsx: Read, Write and Edit XLSX Files. 2020.

5. Yoshida K. tableone: Create “Table 1” to Describe Baseline Characteristics. 2020.

6. Murali AR, Kotwal V, Chawla S. Chronic hepatitis E: A brief review. World J Hepatol. Baishideng Publishing Group Co; 2015;7:2194–201.

***Supplemental Table-ST1. – HEV PCR and Genotype***

|  | | **All HEV-IgM(+) or PCR tested, incl. PCR(-)**  **n = 191** | **Isolated HEV-IgM(+) , incl. PCR(-)**  **n = 125** | **Non-severe acute hepatitis,**  **incl. PCR(-)**  **n = 24** | **Severe hepatitis w/o LD, incl. PCR(-)**  **n = 23** | **Severe hepatitis w LD, incl. PCR(-)**  **n =19** | **p - value** |
| --- | --- | --- | --- | --- | --- | --- | --- |
| **PCR tested** | | 58 (30.4%) | 4 (3.2%) | 19 (79.2%) | 19 (82.6%) | 17 (89.5%) |  |
| **HEV PCR quant. amongst (+), IU/ mL, median [IQR]** | | 4400000 [2298000] | 2200800 [2199200] | 90345 [89655] | 6300000 [2280000] | 392500 [357500] | 0.137 |
| **PCR HEV (+)** | | 19 (30.6%) | 3 (75.0%) | 2 (10.5%) | 7 (36.8%) | 7 (43.8%) |  |
| PCR HEV (-) | | 39 (67.2%) | 1 (25.0%) | 17 (89.5%) | 12 (63.2%) | 10 (62.5%) |  |
| **HEV Genotype available** | | 7 (36.8%) | 0 | 0 | 2 | 5 | 1 |
|  | GT-1, n | 5 (71.4%) | 0 | 0 | 1 (50.0%) | 4 (80.0%) |  |
|  | GT-3, n | 2 (28.6%) | 0 | 0 | 1 (50.0%) | 1 (20.0%) |  |

*Supplemental Table-ST1. – HEV PCR and Genotype results. p-value comparing non-severe acute HEV vs. severe HEV w/o LD vs. HEV w LD*

***Supplemental Table-ST1 Abbreviations***

*(-), negative*

*(+), positive*

*GT, genotype*

*HEV, Hepatitis E virus*

*IgM, immunoglobulin M*

*IQR, inter quantile range*

*IU, International Units*

*n, number*

*PCR, polymerase chain reaction*

*quant., quantitative*

*vs., versus*

*w LD, with liver dysfunction*

*w/o LD, without liver dysfunction*

***Supplemental Table-ST1. – Baseline characteristics of isolated HEV IgM (+)***

|  | | Isolated HEV-IgM(+),  n = 124 | |
| --- | --- | --- | --- |
| Age, years [IQR] | | 51.5 | [28] |
| Sex, n (%) | |  |  |
| Male | | 66 | (53.2%) |
| Female | | 58 | (46.8%) |
|  | **Laboratory values at diagnosis** | | |
| Dg Hb, g/ dL, median ± SD | | 12.7 | ± 2.2 |
| Dg PLT, G/ L, median [IQR] | | 229 | [128] |
| Dg WBC, G/ L, median [IQR] | | 6.6 | [3.5] |
| Dg Na, mmol /L, median [IQR] | | 139 | [4] |
| Dg Creatinine, mg/dL, median [IQR] | | 0.86 | [0.32] |
| Dg Bilirubin, mg/dL, median [IQR] | | 0.6 | [0.5] |
| Dg Albumin, g/L, median [IQR] | | 41.9 | [8.6] |
| Dg ALP, U/L, median [IQR] | | 82 | [46] |
| Dg AST, U/L, median [IQR] | | 28 | [15] |
| Dg ALT, U/L, median [IQR] | | 28 | [26] |
| Dg gGT, U/L, median [IQR] | | 37 | [74] |
| Dg NH3, mmol/ L, median [IQR] | | 34.2 | [40.9] |
| Dg INR, median [IQR] | | 1.1 | [0.2] |

*Supplemental* Table-ST1. Baseline characteristics of isolated HEV IgM (+)

***Supplemental* Table-ST1 Abbreviations**

ALP, Alkaline phosphatase

ALT, Alanine transaminase

AST, Aspartate aminotransferase

CP points, Child-Pugh Points

Dg, Diagnosis

gGT, Gamma-glutamyltransferase

Hb, Hemoglobin

INR, international normalized ratio

IQR, Interquartile Range

Na , Sodium

PLT, Platelets

SD, Standard deviation

St.p., Status post

WBC, White blood cells

***Supplemental Table-ST2. Coinfection with HIV, HAV, HBV +/- HDV and HCV***

|  | **HIV** | **HAV** | **HBV** | **HCV** |
| --- | --- | --- | --- | --- |
| **Severe HEV w/o LD** | 0 ^na2^ | 0 ^na1^ | 0 ^na1^ | 0 ^na1^ |
| **Severe HEV w LD** | 0 ^na1^ | 0 ^na1^ | 0 | 0 ^na1^ |

*Supplemental Table-ST2. – Rates of coinfection with HIV, HAV, HBV +/- HDV and HCV*

***Supplemental Table-ST2* Abbreviations**

HAV, hepatitis A virus

HBV, hepatitis B virus

HCV, hepatitis C virus

HIV, human immunodeficiency virus

na1, NA in 1

na2, NA in 2.

**Supplemental Table-ST3.1. Year of HEV diagnosis (first IgM (+)) or first negative test.**

| **Year** | **HEV-IgM/ PCR tested patients,**  **n** | **HEV-IgM (+) all,**  **n (% of all tested)** | | **HEV-IgM/ PCR (+)**  **n (% of all tested)** | | **Isolated HEV-IgM (+),**  **n (% of all HEV-IgM/ PCR (+))** | |
| --- | --- | --- | --- | --- | --- | --- | --- |
| 2008 | 111 | 9 | (8.1%) | 9 | (8.1%) | 8 | (88.9%) |
| 2009 | 125 | 10 | (8.0%) | 10 | (8.0%) | 8 | (80.0%) |
| 2010 | 111 | 12 | (10.8%) | 12 | (10.8%) | 8 | (90.0%) |
| 2011 | 183 | 10 | (5.5%) | 10 | (5.5%) | 7 | (70.0%) |
| 2012 | 293 | 22 | (7.5%) | 23 | (7.8%) | 20 | (87.0%) |
| 2013 | 687 | 12 | (1.8%) | 13 | (4.4%) | 11 | (84.6%) |
| 2014 | 1089 | 13 | (1.2%) | 14 | (1.3%) | 11 | (78.6%) |
| 2015 | 1270 | 8 | (0.63%) | 8 | (0.63%) | 7 | (87.5%) |
| 2016 | 1737 | 14 | (0.81%) | 16 | (0.92%) | 13 | (81.3%) |
| 2017 | 1981 | 25 | (1.3%) | 25 | (1.3%) | 20 | (80.0%) |
| 2018 | 1358 | 11 | (0.81%) | 11 | (0.81%) | 11 | (100%) |
| **Sum** | **8945** | **146** | **(1.6%)** | **151** | **(1.7%)** | **124** | **(82.1)** |

Supplemental Table-ST3.1. Year of HEV diagnosis (first IgM (+)) or first negative test (supplemental for figure 2)

| **Year** | **Non-severe acute HEV infection**  **n (% of all HEV-IgM/ PCR (+))** | | **Severe HEV w/o LD,**  **n (% of all HEV-IgM/ PCR (+))** | | **Severe HEV w LD,**  **n (% of all HEV-IgM/ PCR (+))** | | **Severe HEV - overall** | |
| --- | --- | --- | --- | --- | --- | --- | --- | --- |
| 2008 | 0 | (0%) | 0 | (0%) | 1 | (11.1%) | 1 | (11.1%) |
| 2009 | 0 | (0%) | 2 | (20.0%) | 0 | (0%) | 2 | (20%) |
| 2010 | 0 | (0%) | 2 | (16.7%) | 2 | (16.7%) | 4 | (33.4%) |
| 2011 | 2 | (20.0%) | 0 | (0%) | 1 | (10%) | 1 | (10.0%) |
| 2012 | 1 | (4.35%) | 1 | (4.4%) | 1 | (4.4%) | 2 | (8.7%) |
| 2013 | 0 | (0%) | 1 | (7.7%) | 1 | (7.7%) | 2 | (15.4%) |
| 2014 | 1 | (7.1%) | 0 | (0%) | 2 | (14.3%) | 2 | (14.3%) |
| 2015 | 1 | (12,5%) | 0 | (0%) | 0 | (0%) | 0 | (0%) |
| 2016 | 2 | (12,5%) | 1 | (6.3%) | 0 | (0%) | 1 | (6.3%) |
| 2017 | 0 | (0%) | 4 | (16%) | 1 | (4.0%) | 5 | (20.0%) |
| 2018 | 0 | (0%) | 0 | (0%) | 0 | (0%) | 0 | (0%) |
| **Sum** | **7** | **(4.6%)** | **11** | **(7.3%)** | **9** | **(6.0%)** | **20** | **(13.2%)** |

**Supplemental Table-ST3.2. Year of HEV diagnosis (first IgM (+)) or first negative test**

Supplemental Table-ST3 Year of HEV diagnosis (first IgM (+)) or first negative test (supplemental for figure 2)

**Supplemental Table-ST3 Abbreviations**

(+), positive

ALT, alanine transaminase

HEV, Hepatitis E virus

IgM, immunoglobulin M

PCR, polymerase chain reaction

ULN, upper limit of normal

**Supplemental Table-ST4. – Male to female ratio per year among severe HEV infection.**

| **Year** | **Male: Female ratio** | |
| --- | --- | --- |
| 2008 | 2 | 6:3 |
| 2009 | 0.43 | 3:7 |
| 2010 | 2 | 8:4 |
| 2011 | 2.33 | 7:3 |
| 2012 | 0.77 | 10:13 |
| 2013 | 1.6 | 8:5 |
| 2014 | 2.5 | 10:4 |
| 2015 | 3 | 6:2 |
| 2016 | 2.2 | 11:5 |
| 2017 | 0.67 | 10:15 |
| 2018 | 0.84 | 5:6 |

Supplemental Table-ST4. Male to female ratio, for IgM (+) patients. Supplemental to Figure 3.

**Supplemental Table-****ST5. Characteristics of patients with severe HEV infection**

| **Year** | **Severe HEV** | **Underlying liver Disease (Y)** | **Cirrhosis** | **Travel history** | **Immunosuppression,**  **HIV (+),**  **Organ Tx** | **HEV-RNA (IU/mL) / (+)** | **HEV-GT** | **Peak ALT (U/L)** | **Peak Bili (mg/dL)** | **Ascites (Y/N)** | **HE (Y/N)** | **Admitted (Y/N)** | **ICU (Y/N)** | **Outcome (30d)** |
| --- | --- | --- | --- | --- | --- | --- | --- | --- | --- | --- | --- | --- | --- | --- |
| 2012 | w LD | N | N | India | N | (+) | 1 | 3465 | 23.31 | N | N | Y | N | Bilirubin decreased to 4,2 mg/dl, ALT normalized |
| 2017 | wo LD | N | N | N | NTX | 1500000 | - | 1056 | 3.53 | N | N | N | N | Resolved |
| 2016 | wo LD | N | N | Thailand | N | 69000 | 3 | 2101 | 2.18 | N | N | N | N | Resolved |
| 2011 | w LD | N | N | Greece & Turkey | N | (+) | - | 3287 | 9.11 | N | N | Y | N | Resolved |
| 2010 | wo LD | N | N | - | EBV (+) | (+) | - | 223 | 1,07 | - | - | - | - | Resolved |
| 2009 | wo LD | N | N | India, 2 Tattoos | N | (+) | - | 1887 | 4,96 | N | N | Y | N | Resolved |
| 2017 | wo LD | N | N | N | HTX | 6300000 | - | 185 | 1.19 | N | N | N |  | Chronic HEV; Ribavirin – Relapse but TA normalized |
| 2010 | w LD | N | N | India | N | (+) | - | 1751 | 7.1 | N | N | Y | N | Resolved |
| 2008 | w LD | N | N | India | N | (+) | - | 3143 | 15.91 | N | N | Y | N | Resolved |
| 2017 | w LD | NASH | N | N | DM II, F2. | 750000 | 3 | 2539 | 9.84 | N | N | Y | N | Resolved |
| 2017 | w LD | N | N | N | Concomitant bacterial infection. DM II | 920000 | - | 2089 | 1.73 | N | N | Y |  | Lost to FU |
| 2013 | wo LD | N | N | N | N | (+) | - | 1293 | 0.89 | N | N | N | N | Remission |
| 2014 | w LD | N | N | India & Nepal | N | 35000 | 1 | 1322 | 12.00 | N | N | N | N | Remission |
| 2014 | w LD | N | N | India, Sri Lanka | N | (+) | 1 | 3980 | 15.67 | Y | N | Y | N | Lost to FU |
| 2010 | w LD | - | - | - | - | (+) | 1 | 3098 | 5.81 | N | - | - | - | Lost to FU |
| 2012 | wo LD | N | N | N | Mb. Hashimoto, acute EBV | (+) | - | 545 | 4.18 | N | N | Y | N | Remission |
| 2009 | wo LD | N | N | N | N | (+) | - | 1310 | 1.27 | N | N | Y | N | Remission |
| 2010 | wo LD | N | N | - | N | (+) | - | 1548 | 2.17 | N | N | Y | N | Remission |
| 2017 | w LD | N | N | India | N | 3200000 | 1 | 3091 | 6.2 | N | - | - | - | Remission |
| 2013 | w LD | N | Y | - | DM, Lung-Tx (Immunosuppression) | (+) | - | 2162 | 23.76 | Y | N | Y | N | Prolonged HEV viremia (5 months), Remission. |

Supplemental Table-ST5. Recorded Extrahepatic manifestations.

**Supplemental Table-ST5 Abbreviations**

(+), positive

(N), No

(Y), Yes

d, day

DM, diabetes mellitus

GT, genotype

HEV, hepatitis E virus

HIV, human immunodeficiency virus

Tx, transplantation

w LD, with liver dysfunction

wo LD, without liver dysfunction

***Supplemental Table-ST6. – Extrahepatic manifestations recorded***

|  | **All HEV-IgM or PCR(+),**  **n = 151** | **Isolated HEV-IgM(+)**  **n = 131** | **Non-severe acute HEV,**  **n = 7** | **Severe HEV w/o LD,**  **n = 11** | **Severe HEV w LD,**  **n =9** |
| --- | --- | --- | --- | --- | --- |
| **Severe Thrombocytopenia (PLT ˂ 50 G/L)** | 2 (1.32%) | 2 (1.53%) | 0 | 0 | 0 |
| **Anaemia (Hb ˂12 mg/dL)** | 8 (5.3%) | 3 (2.3%) | 3 (42.9%) | 2 (18.2%) | 0 |
| **Haemolytic anaemia** | - | - | 0 | 0 | 0 |
| **Acute Kidney injury** | - | - | 0 | 0 | 0 |
| **Acute pancreatitis** | - | - | 0 | 0 | 0 |
| **Neurological Symptoms** | - | - | 0 | 0 | 0 |

*Supplemental Table-ST6.* *Extrahepatic manifestations recorded.*

***Supplemental Table-ST6 Abbreviations***

*(+), positive*

*HEV, hepatitis E virus*

*n, number.*

*PLT, Platelet count*

*w LD, IgM, Immunoglobulin M*

*w/o LD, without liver dysfunction*

***Supplemental figure-FS1. Time course of gGT and ALP amongst patients with severe HEV***

Supplemental Figure-FS-1. The time course of A) ALP and B) gGT.

**Supplemental Figure-FS-1 Abbreviations**

*ALP, Alkaline Phosphatase*

*ALT, Alanine Transaminase*

*d, day.*

*gGT, Gamma-glutamyl Transferase*
